# Shorter telomeres precede population extinction in wild lizards

**DOI:** 10.1038/s41598-017-17323-z

**Published:** 2017-12-05

**Authors:** Andréaz Dupoué, Alexis Rutschmann, Jean François Le Galliard, Jean Clobert, Frédéric Angelier, Coline Marciau, Stéphanie Ruault, Donald Miles, Sandrine Meylan

**Affiliations:** 1 CNRS UPMC, UMR 7618, iEES Paris, Université Pierre et Marie Curie, 4 place Jussieu, 75005 Paris, France; 2 Station d’Ecologie Théorique et Expérimentale du CNRS à Moulis, UMR 5321, 09200 Saint Girons, France; 3 School of Biological Sciences, University of Auckland, Auckland, New Zealand; 4 Ecole Normale Supérieure, PSL Research University, CNRS, Centre de recherche en écologie expérimentale et prédictive (CEREEP-Ecotron IleDeFrance), UMS 3194, 78 rue du château, 77140 Saint-Pierre-lès-Nemours, France; 5 CNRS CEBC-ULR, UMR 7672, Villiers en Bois, 79360 Beauvoir sur Niort, France; 6 Department of Biological Sciences, Ohio University, Athens, OH 45701 USA; 7 ESPE de Paris, Université Sorbonne Paris IV, 10 rue Molitor, 75016 Paris, France

## Abstract

Identifying the early warning signals of catastrophic extinctions has recently become a central focus for ecologists, but species’ functional responses to environmental changes remain an untapped source for the sharpening of such warning signals. Telomere length (TL) analysis represents a promising molecular tool with which to raise the alarm regarding early population decline, since telomere attrition is associated with aging processes and accelerates after a recurrent exposure to environmental stressors. In the southern margin of their range, populations of the common lizard (*Zootoca vivipara*) recently became extinct at lowest elevations due to changes in climate conditions. However, the proximal signals involved in these demographic declines are still unknown. Here, we sampled 100 yearling lizards from 10 natural populations (n = 10 per population) along an extinction risk gradient. Relative lizard abundance dramatically dropped over 12 years in low-altitude populations characterized by warmer ambient temperatures and higher body growth of lizards early in life. A non-linear relationship was found between TL and population extinction risk, with shorter telomeres in populations facing high risk of extinction when compared to non-threatened ones. Our results identify TL as a promising biomarker and imply that population extinctions might be preceded by a loop of physiological aging.

## Introduction

Anthropogenic activities lead to regime shifts and critical transitions in ecological systems at an unprecedented pace, including extinctions of an increasing number of species now and for the future^1,2^. In deteriorating environments, tipping points often precede the demographic collapses of these species, and the development of early warning signals of these tipping points has recently become a central focus for ecologists^3–7^. Tipping points have been modelled in single species systems and examined with laboratory experiments^4,6^, in which extinctions are preceded by a critical slow down – that is, a decreasing rate of recovery of population size after a small perturbation. However, the universality of this early warning signal has been questioned and our ability to detect this demographic phenomenon in complex and resilient natural systems may be very low^5^. In foreseeing the dynamics of population extinction, species’ functional responses to environmental stressors are also a critical prerequisite to consider^8^. There is an urgent need to characterize the early functional changes and proximal signals preceding population extinction, and the search for reliable physiological biomarkers has therefore become a major quest for ecological studies of global change-induced extinctions^9–11^.

Telomeres are repetitive non-coding DNA sequences (e.g. TTAGGG in vertebrates) associated with nucleoproteins that cap the end of eukaryotic chromosomes^12,13^. At each cell division, telomere length (TL) generally decreases during DNA replication and oxidative damages can accelerate telomere loss, but TL can also be restored by telomerase activity^12,14^. Telomeres are essential for chromosomal integrity, and as such telomeres that are too short lead to cell apoptosis, genome instability, and whole-organism aging^15^. Harmful or stress-inducing environments caused for example by carcinogenic compounds, pollutants, thermal stress, or pathogens accelerate telomere erosion *in vivo* as demonstrated in wild species and humans^16–19^. At the individual level, TL is usually positively linked to survival probability in wild animals^20–22^, although this relationship is not systematic^23^. Besides, TL may also negatively correlate with the reproductive performances in vertebrates^24,25^. Thus, TL should provide a relevant biomarker of fitness in relation to environmental stressors^26,27^. Notably in ectotherms, TL correlates with thermal stress experienced by natural populations^28,29^, and population extinction due to climate warming may therefore be signalled by telomere loss.

In response to global warming, ectotherms are particularly exposed to extinction risk because of their reliance on external heat sources to raise their body temperatures and the thermal sensitivity of their physiological and demographic performance^30–33^. A significant proportion of local lizard populations have been extirpated by climate warming worldwide during the last three decades^32,34,35^. This is especially true in areas such as mountain or tropical ecosystems that associate strong endemism, and in which species are adapted to narrow thermal ranges^36,37^. For instance, mountain populations of the common lizard (*Z. vivipara*) recently went extinct at low altitudes in the southern, hot margin of the European range, and this has been directly correlated with a higher frequency of spells of warm and dry weather during the last two decades^32,34^. The Common lizard is a perfect example of a cold-adapted species, although the thermal preferences may vary along altitude^38^. Low altitude environments involve more frequent exposures to abnormally high thermal conditions^32,34,36^, and therefore this species is particularly interesting to study the functional responses to warming conditions. Increased temperatures benefit individual lizards in early life thanks to a faster body growth rate and earlier sexual maturation, while inducing delayed costs on survival and longevity during the hottest years, which eventually leads to demographic collapses^34,39,40^.

We took advantage of a comparative context in this species to investigate the variations of TL across natural populations that differ in environmental conditions and associated extinction risk. During the late spring activity season in May-June 2015, we sampled 100 yearling lizards (i.e., 10–11 months old) from 10 natural populations of *Z. vivipara* (n = 10 per populations) in the southern margin of the species distribution in the Massif Central mountain range in France. Our samples included populations that are distributed at different altitudes and some of which are close to extinction. We hypothesised that TL would correlate with population risk of extinction and predicted that shorter telomere should indicate population collapse.

## Results

**Figure 1:**
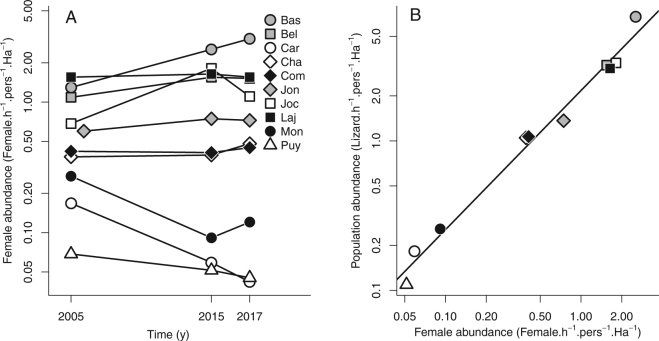
Changes in the abundance of the common lizard from monitored populations (Barnesac: Bas, Bel Air: Bel, M^t^ Caroux: Car, Chalet du M^t^ Lozère: Cha, Col du Cheval Mort: Com, Gerbier de Jonc: Jon, Gerbier de Jonc II: Joc, Lajo: Laj, Montselgues: Mon, and Puy Mary: Puy) over 12 years. We used (**A**) the change in adult female abundance to estimate the change in population abundance including adults females and males and yearlings because (**B**) both are highly correlated in 2015 (*r* ^2^ = 0.96, *P* < 0.001).

**Figure 2:**
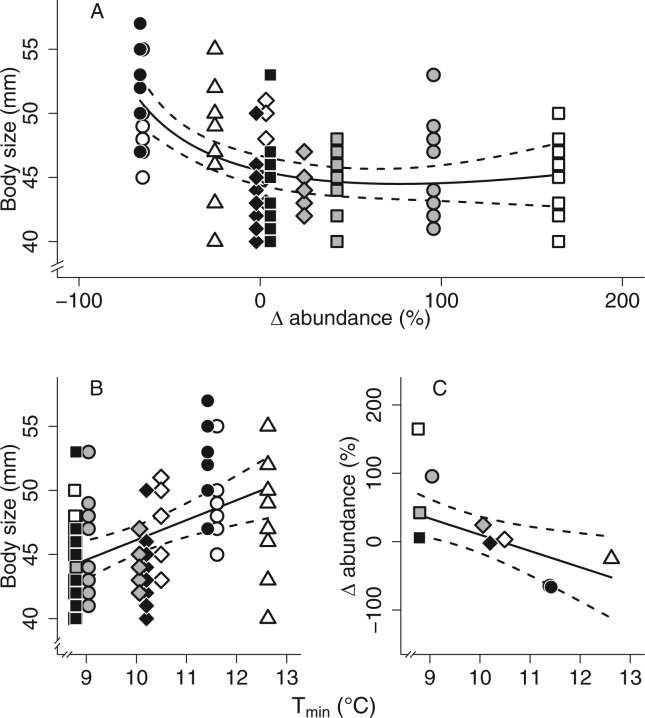
Natural context of population collapse in the studied populations of common lizards. (**A**) Yearlings (n = 100) grew to a larger body size (snout-vent length) in collapsing populations than in stable or increasing populations (Bas: grey circle, Bel: grey square, Car: white circle, Cha: white diamond, Com: black diamond, Jon: grey diamond, Joc: white square, Laj: black square, Mon: black circle, and Puy: white triangle). The lizard body size at the yearling stage (**B**) was positively related to minimal local temperature values (average *T*_min_, during the night), and (**C**) the relative changes in lizard abundance were negatively related with *T*_min_. In each graph, the predictions of the selected model were fitted on the data (solid line) together with the 95% confidence interval (dashed lines).

Our study populations have been monitored since 2005 and we estimated the relative changes in lizard abundance over 12 years (Fig. 1, supplementary Table S1), as well as local thermal conditions during the survey including minimal (*T*_min_) and maximal (*T*_max_) daily temperatures. We used relative changes in abundance (*r*) to determine the IUCN category of populations. We identified two populations with very high risk of extinction and one with high risk of extinction, while the remaining populations were non-threatened (supplementary Table S1). Body size of yearlings (i.e., an index of body growth early in life) decreased non-linearly with relative change in abundance, since lizards from the collapsing populations had a larger body size (Fig. 2A, supplementary Table S2). In addition, both the lizard body size and the relative changes in abundance were positively and negatively related to *T*_min_, respectively (Fig. 2B,C, supplementary Tables S2 and S3). Overall, lizards also had bigger body size in populations located at the lowest altitudes (supplementary Table S2). To rank populations along a single, common axis of extinction risk, we computed variables best describing each population (relative changes in abundance, *T*_min_, and altitude) together in a principal component analysis (as detailed in supplementary Table S4). The first component (PC_1_), which explained 69.5% of the variation, was negatively correlated with relative change in abundance (inertia: 40.3%) and altitude (inertia: 24.3%), and positively correlated with *T*_min_ (inertia: 35.4%). It thus provided a composite score related to extinction risk.

**Figure 3:**
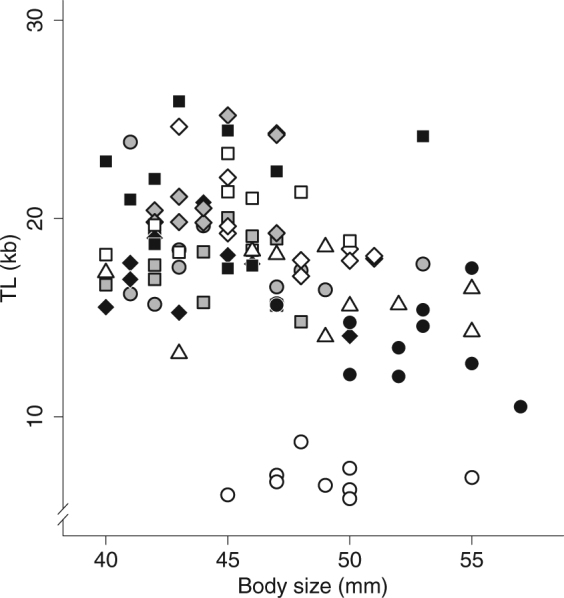
Lack of relationship between telomere length (TL) and lizard body size. TL was not correlated with lizard body size (*P* = 0.200) in wild populations (Bas: grey circle, Bel: grey square, Car: white circle, Cha: white diamond, Com: black diamond, Jon: grey diamond, Joc: white square, Laj: black square, Mon: black circle, and Puy: white triangle).

TL differed strikingly among populations (relative inter-population coefficient of variation: 25.9%; F_9,79_ = 13.39, *P* < 0.001), revealing a wide range of TL (5.87–25.91 kb) in wild individuals of the same age class. In contrast, inter-individual variation within populations was relatively low and homogeneous (mean intra-population coefficient of variation: 14.7%; homoscedasticity test of Bartlett: *P* = 0.867). TL was not correlated with lizard body size (F_1,79_ = 1.67, *P* = 0.200, Fig. 3), and was not different between males and females (F_1,79_ = 1.06, *P* = 0.307) irrespective of the study population (population × sex interaction: F_9,79_ = 0.66, *P* = 0.744).

**Figure 4:**
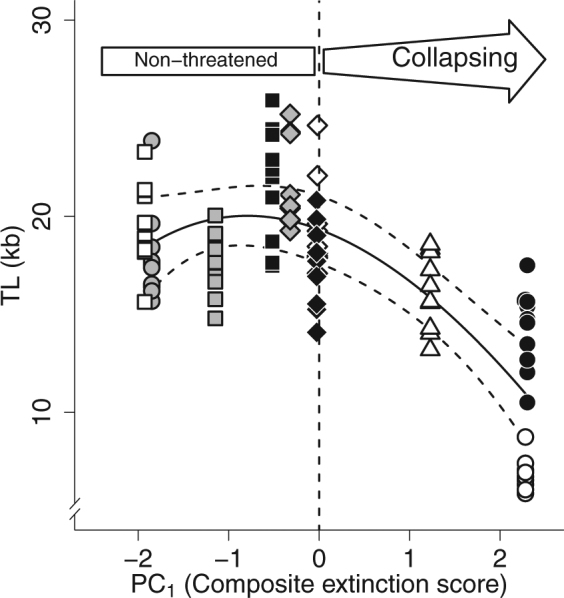
Non-linear relationship between telomere length (TL) and population extinction risk score. A population extinction risk score was assessed from the first axis (PC_1_) of a principal component analysis, including the main determinant of population collapse (the relative change of abundance, *T*_min_, and altitude). For PC_1_ < 0, the populations are non-threatened, and when PC_1_ > 0, the risk of collapsing progressively increases (Bas: grey circle, Bel: grey square, Car: white circle, Cha: white diamond, Com: black diamond, Jon: grey diamond, Joc: white square, Laj: black square, Mon: black circle, and Puy: white triangle). The predictions of the selected model were fitted on the data (solid line) together with the 95% confidence interval (dashed lines). A similar pattern and result was obtained when relative change in abundance (Δabundance) was used as a covariate instead of PC_1_ score.

According to the model selection approach, inter-population variation in TL was better explained by the PC_1_ score of the population followed closely by altitude and next by relative changes in abundance (Table 1). In the best supported model, TL was non-linearly related with the population extinction risk score (Fig. 4, Table 1). In populations of least concern, TL was similarly high across sites (TL = 19.31 ± 2.71 kb), whereas it was shorter in the two populations experiencing the most significant demographic declines (TL = 10.81 ± 4.00 kb). In comparing the influence of PC_1_ on TL or SVL, we found that PC_1_ explained higher degree of variation in TL (AICc: 480.61, *r* ^2^ = 0.68) than SVL (AICc: 534.54, *r* ^2^ = 0.34).

**Table 1:** AICc based model selection comparing a null model (intercept only) to models testing linear and non-linear (logarithmic) relationships between telomere length and computed environmental and individual variables (PC_1_ – first axis of a principal component analyse including relative change in abundance, *T*_min_, and altitude) on telomere length in yearling common lizards (*n* = 100). In models, the relative change in abundance and PC_1_ were added a constant to enable log transformation. Population was treated as a random factor in all models to account for intra-population variation and non-independence.

Model	k	AICc	ΔAICc	*w*_*i*_	*β* (±SE)	Log likelihood
PC_1_ + log(PC_1_)	5	480.61	0.00	0.39	−21.5 ± 6.3	−234.99
					198.0 ± 63.8	
Altitude + log(altitude)	5	481.61	1.00	0.24	−0.3 ± 0.1	−235.49
					356.6 ± 115.4	
Δabundance + log(abundance)	5	482.75	2.14	0.14	−0.07 ± 0.03	−236.06
					11.4 ± 3.2	
PC_1_	4	483.04	3.15	0.08	−1.7 ± 0.4	−237.31
*T*_max_ + log(*T*_max_)	5	483.76	4.05	0.05	−5.6 ± 3.4	−236.56
					213.3 ± 113.2	
*T*_max_	4	484.66	4.68	0.04	0.9 ± 0.2	−238.12
Altitude	4	486.25	5.64	0.02	0.02 ± 0.01	−238.92
SVL_mean_	4	487.85	7.23	0.01	−0.9 ± 0.4	−239.71
*T*_min_	4	488.02	7.41	0.01	−1.9 ± 0.8	−239.80
Δabundance	4	489.04	8.43	0.01	0.03 ± 0.02	−240.31
SVL_mean_ + log(SVL_mean_)	5	489.90	9.29	0.00	4.4 ± 13.4	−239.63
					−253.6 ± 636.7	
*T*_min_ + log(*T*_min_)	5	490.24	9.63	0.00	−2.0 ± 14.3	−239.80
					1.5 ± 148.7	
*Null* (intercept)	3	490.71	10.09	0.00	17.3 ± 1.2	−242.23

k: number of parameters, ΔAICc: difference with AICc of the best model, *w*_*i*_: model likelihood.

## Discussion

We found that populations at the lowest altitudes and exposed to the highest daily temperatures during our survey were characterized by a strong relative loss of lizard abundance during the last 12 years. This correlation between population decline and climate conditions is in accordance with results found in an earlier study spanning multiple mountain ranges of the southern margin of this species in Europe^36^. Yearling lizards from the collapsing populations attained bigger size, which is also consistent with long-term warming trends in one of our study population and recent experiments in field enclosures showing that increased ambient temperatures improve the growth rate and body size early in life^39^, but are associated with stronger risks of demographic collapse^32,40^. These results should be interpreted in regards with sample size; that is although we sampled a consequent number of individuals (n = 100), we still have a limited representation of collapsing populations (n = 30 individuals from 3 populations). Bigger body sizes are probably attained thanks to higher temperature facilitating longer activity periods or higher individual growth rates, but we cannot exclude that warmer environments also selected for faster body growth in this species^34,39^. More generally, the acceleration of a species pace of life can also lead to dramatic changes in the community structure, before their extinction, which may contribute to observed patterns.

Remarkably, we found that yearling individuals showed decreasing TL with extinction risk irrespectively of their sex probably because both males and females are non-reproductive and have similar physiological requirements at the yearling stage in these populations^41^. The degree of variation between populations was unexpectedly high, hence suggesting significant dynamic of telomere erosion early in life within populations. Mechanisms underlying intra-specific variation in TL are poorly known in reptiles and shorter telomeres in yearling lizards from collapsing populations may result from multiple, overlapping causes. Individuals could have inherited shorter telomeres from their parents (population genetic differences) and besides, telomeres may have been shortened during embryonic life through maladaptive maternal effects^42–45^. In support for differential genetic population structure, populations are distant and isolated from each other, especially in the collapsing populations (i.e., top of mountains or volcano crater) that are associated with few possibility for dispersal. Besides, dispersal may be inhibited by climate change, therefore suggesting a rapid homogenization of the genetic profile within collapsing population^46^. Furthermore, TL may decrease after birth faster early in life either as a result of direct effects of increased thermal stresses and costs of a faster juvenile growth^29^. The dominant effect of the composite extinction score on TL and a good degree of support for the alternative models, including other environmental covariates (Table 1), advocates for a potential multi-factorial determinism of TL reduction in collapsing populations. Additional fieldwork and targeted experiments are required to identify causal mechanisms underlying observed geographic patterns of TL variation.

It is worth noting that TL might have been influenced by a change in body growth rate across populations^47,48^, independently of population collapse. This alternative hypothesis is unlikely since higher body growth rate in warmer climate conditions has been demonstrated to be correlated with a lower annual survival rate driving the long-term population collapse in the common lizard^40^. Moreover, when comparing the contribution of PC_1_ *versus* the one of body size on lizard TL, we found a better support for an effect of PC_1_ on TL and we did not find any relationship between TL and individual body size, hence suggesting climatic effects on TL independent of growth. Altogether, our results suggest that even if change in climatic conditions may induce higher body growth rate, population extinction risk was a better descriptor of variation in yearling TL than SVL.

The consistent influence of *T*_min_ on relative change in abundance, lizard body size, and PC_1_ suggests that warmer air conditions at night, when lizards are at rest in their burrows, was a major contributing factor. Cold-adapted ectotherms such as the common lizard adopt different strategies when experiencing low temperatures, but this usually translates into a lower locomotor and physiological activity and eventually retreat into a refuge^49,50^. Warmer thermal conditions during these periods of low activity cannot be behaviourally avoided through thermoregulation, which likely results in a higher resting metabolic rate and consequently a higher production of oxidative damages^51^, which is well-known to accelerate telomere attrition^14^. In order to clarify the contribution of the multiple causes driving telomere erosion before population collapse, further investigations are required with more populations, pedigree-based studies and common garden experiments.

Identifying reliable biomarkers preceding imminent extinction is urgently needed since early demographic signals, such as a critical slow down, require long-term demographic data and are often not within the reach of most conservation and ecological studies^9–11^. TL as a biomarker of population extinction risk might also be relevant in endotherms. However, in endotherms, climate change may induce physiological stress through indirect factors such as dramatic changes in resource availability or habitat quality^52,53^, which may in return accelerate the rate of telomere attrition^16^. Besides, it is noteworthy that the TL can be elongated thanks to telomerase activity^54^, therefore suggesting a potential key function of this enzyme on aging process. Overall, further studies are critically required to investigate the potential for TL to correlate with population dynamics. Here, we found that strong inter-population variation in TL was negatively associated with population extinction risks caused by multifactorial changes in environmental conditions along an altitudinal gradient. Therefore we identified TL as one critical biomarker of the population extinction risk, and propose that TL likely constitutes the most reliable early warning signal to date to objectively assess population state in the wild. All individuals from this study were in the same chronological age class, and yet our results suggest that yearlings from collapsing populations may be biologically already old before reproducing^13^. Offspring TL in early life may correlate with longevity^20^, therefore our results imply that population extinctions are likely preceded by an irreversible loop of physiological aging.

## Methods

### Studied populations and environmental variables

Populations of *Z. vivipara* were sampled in the Massif Central region in France (i.e., viviparous form) during the same activity season in May-June 2015. In each population, we estimated abundance by the number of captured lizards standardized to the time spent searching for lizards in each population, the number of persons capturing lizards and area of the study population (Fig. 1, supplementary Table S1). Populations are typically monitored under the same climatic conditions so that abundance estimations are comparable between and within populations. All populations were initially sampled in 2005 to analyse geographic variation in reproduction phenology and life history strategies of reproduction, and therefore only the initial abundances of adult females were available (Fig. 1A). We used the same method to measure the changes in adult female abundance to assess the changes in population abundance between 2015 and 2005 because both are highly correlated (*r* ^2^ = 0.96, *P* < 0.001, Fig. 1B). In addition, females make up a dominant proportion of the adult population, which implies that demographic comparisons are more reliable with this life stage. In each population, we also recorded air temperature every 30 min over 3 weeks (from 29^th^ June to 17^th^ July 2015) using 2 or 3 sensors per population (Thermochrons©, Maxim Integrated Products, Sunnyvale, CA, USA) placed in microhabitats used by lizards. We considered averages of daily minimal and maximal temperatures to describe the thermal conditions of the populations.

All methods were performed in accordance with laws relative to capture, transport and experiments on *Zootoca vivipara* (DREAL Languedoc Roussillon permit #2013-274-0002, DREAL Midi-Pyrénées permit #81-2013-05, and DREAL Auvergne, permit #2013/DREAL/259).

### Sampling procedures and telomere assays

We captured 10 yearling lizards in each population (n = 100) from late May to late June 2015, and within 3 minutes after their capture, lizards were bled (whole blood volume: 40 µl) from the postorbital sinus and then measured (snout-vent length, SVL ± 1 mm). The day of capture, blood samples were maintained on ice in a cooler below 6 °C. In laboratory, samples were centrifuged at 11 000 rpm for 5 min, plasma was separated from red blood cells and all samples were kept frozen at −28 °C until lab assays. We determined TL at the Centre d’Etudes Biologiques de Chizé (ULR, UMR 7372, France) using the Telomere Restriction Fragment method following methods as previously described^18^. Telomere length was obtained from Southern blot using the TeloTAGGG Telomere Length Assay (Roche, Mannheim, Germany). 10 µL of red blood cells were digested in proteinase K, and DNA was purified using DNeasy blood and tissue kit (Qiagen). The quality and the amount of extracted DNA was quantified by optical density spectrophotometry (NanoDrop). We optimized the DNA amount to 0.7 µg of DNA per sample thanks to previous investigations^18^. Telomeric DNA from each sample was isolated using *Hinfl* and *RsaI* restriction enzymes for 16 h at 37 °C. We used a pulse-field gel electrophoresis to (Bio-Rad) on a 0.8% agarose gel for DNA migration. We used 5 gels to run the 100 samples, and each sample was randomly assigned to a gel. We used the same three samples with two replications on each gel to measure intra-gel and inter-gel variation coefficients (respectively 1.40% and 4.20%). The gels were run at 3.0 Volt.cm^−1^ with an initial switch time of 0.5 s to a final switch time of 7 s for 14 h. After DNA migration, DNA was transferred by capillary action onto a nitrocellulose membrane by Southern blot (Hybond N+, Amersham Life Science, Amersham, UK). DNA was fixed on the membrane during an incubation at 120 °C for 20 min. The DNA was then hybridized with a digoxigenin-labeled probe specific for telomeric sequences and incubated with antidigoxigenin-specific antibody before visualization with a Chemidoc (Bio Rad) allowing an optimal image quality. Telomere length was then determined using ImageJ software to extract telomere smear densities. Lane-specific background was subtracted from each density value and TL (mean value) was then calculated using ImageJ software in a window of 5–21 kb and extrapolating up to 50 kb to include the whole smear^55^ (Supplementary Fig S1). TL assays were successfully achieved on all samples (n = 100) so that the different analyses on TL are then performed on all the individuals.

### Analyses

Statistical analyses were performed with R software (version 3.3.2, R Development Core Team 2016). We first used a linear model to determine the effects of body size (snout-vent length - SVL), populations, sex and their interaction on TL. In this initial model, p-values were obtained from type III sum of squares, and considered significant for α < 0.05. Although the paper focus on TL, initial analyses on relationships between SVL, relative changes in abundance and environmental covariates were performed in order to check the consistence of the natural context with previous experimental demonstration on the population decline under warmer climates^40^. Hence, we then studied the relationships between lizard SVL and the relative change in abundance, *T*_min_, *T*_max_, or altitude using linear mixed models [package nlme^56^], with population set as a random factor to account for intra-population variability and non-independence. Following the model selection with information theoretic approach, we compared the second-order Akaike Information Criterion corrected for small sample size [AICc, package AICcmodavg^57^] of a model including only the intercept (i.e., null model) to models testing linear or non-linear (logarithmic transformation) relationships between lizard SVL and environmental variables (supplementary Table S2). The relative changes in abundance assumed negative values and were therefore added a constant to enable log transformation. We used the same method with linear models to compare the relationship between the relative changes in abundance and population covariates such as *T*_min_, *T*_max_, altitude, and mean lizard SVL (supplementary Table S3). Mean lizard SVL was calculated per population so that we obtained one population estimate of yearling body size. Given that several variables were highly correlated and related to population collapse, we computed the relative changes in abundance, *T*_min_ and the altitude together in a principal component analysis [package ade4^58^] since those were found with the best statistical support in previous analyses (supplementary Tables S2 and S3). We used the first axis (PC_1_) as a composite score of extinction risk since it was mainly determined by the relative changes in lizard abundance (supplementary Table S4). We compared linear mixed models with the model selection approach as described above to test linear or non-linear relationships between TL and population extinction risk or other environmental variables.

## Electronic supplementary material

Supplementary information
