# Supplementary material for: Shorter telomeres precede population extinction in wild lizards
Source: Sci Rep. 2017 Dec 5;7:16976. doi: 10.1038/s41598-017-17323-z (PMC5717062; doi:10.1038/s41598-017-17323-z)

Supplementary Information

Shorter telomeres precede population extinction in wild lizards

Andréaz Dupoué [1] *, Alexis Rutschmann [2,3], Jean François Le Galliard [1,4], Jean Clobert [2], Frédéric Angelier [5], Coline Marciau [1], Stéphanie Ruault [5], Donald Miles [6], Sandrine Meylan [1,7]

Supplementary table S1. Population characteristics including the main environmental covariates related to population extinction risk. Table shows the mean ± SE of minimal and maximal temperature (T_{min} and T_{max} respectively) recorded between June 29 and July 17, 2015. We used the relative change in abundance (r) to assess the index of IUCN conservation status of each population [r = (Abundance^{2015} – Abundance^{2005}) / Abundance^{2005}] [1].

					Abundance			
Population	Altitude (m)	Surface (Ha)	T_{min}	T_{max}	2005	2015	r	IUCN category
BAS	1515	0,8	9.04 ± 0.62	38.50 ± 0.62	1,30	2,53	1,0	LC
BEL	1418	0,9	8.80 ± 0.56	33.96 ± 0.49	1,09	1,55	0,4	LC
CAR	1047	4,0	11,39 ± 0.43	27,31 ± 0.50	0,17	0,06	-0,6	EN
CHA	1429	2,6	10.49 ± 0.77	35.63 ± 0.68	0,38	0,39	0,0	LC
COM	1405	2,4	10.20 ± 0.61	29.93 ± 0.68	0,42	0,41	0,0	LC
JOC	1296	1,5	8.77 ± 0.53	35.78 ± 0.61	0,68	1,81	1,6	LC
JON	1398	1,7	10.06 ± 0.43	33.73 ± 0.68	0,60	0,74	0,2	LC
LAJ	1330	0,6	8.79 ± 0.46	39.04 ± 0.66	1,55	1,64	0,1	LC
MON	1049	4,3	11.42 ± 0.42	33.13 ± 0.32	0,27	0,09	-0,7	EN
PUY	1434	19,4	12.63 ± 0.60	32.55 ± 0.84	0,07	0,05	-0,3	VU

[1] *IUCN Red List Categories and Criteria and Guidelines for Using the IUCN Red List Categories and Criteria. Scenario A2.* Endangered (EN): very high risk of extinction in the wild; Vulnerable (VU): high risk of extinction in the wild; Least Concern (LC): non-threatened populations.

Supplementary table S2. AICc based model selection comparing a null model (intercept only) to models testing linear and non-linear (logarithmic) relationships between lizard body size and the relative changes in population abundance, T_{min}, T_{max}, and altitude in yearling lizards (n = 100). In models, the relative change in abundance was added a constant to enable log transformation. Population was set as a random factor in all models to account for intra-population variation and non-independence.

Model	k	AICc	ΔAICc	w_i	β (± SE)	Log likelihood
Δabundance + log(abundance)	5	536.81	0.00	0.58	0.04 ± 0.02<br>-4.7 ± 2.2	-263.09
T_{min}	4	539.29	2.48	0.17	1.6 ± 0.4	-265.44
Altitude + log(altitude)	5	540.37	3.56	0.10	0.14 ± 0.08<br>-189.7 ± 99.1	-264.87
Altitude	4	541.36	4.55	0.06	-0.010 ± 0.004	-266.47
T_{min} + log(T_{min})	5	541.51	4.69	0.06	2.0 ± 8.5<br>-5.0 ± 87.8	-265.43
Δabundance	4	542.95	6.14	0.03	-0.02 ± 0.01	-267.26
Null (intercept)	3	545.25	8.43	0.01	46.4 ± 0.9	-269.50
T_{max}	4	546.40	9.59	0.00	-0.25 ± 0.24	-268.99
T_{max} + log(T_{max})	5	548.49	11.68	0.00	-1.6 ± 4.0<br>45.3 ± 131.0	-268.93

k : number of parameters, ΔAICc: difference with AICc of the best model, w_i: model likelihood.

Supplementary table S3. AICc based model selection comparing a null model (intercept only) to models testing linear and non-linear (logarithmic) relationships between relative changes in abundance and mean body size (SVL_{mean}), T_{min}, T_{max} and altitude in 10 populations.

Model	k	AICc	ΔAICc	w_i	*β* (± SE)	Log likelihood
T_{min}	4	120.62	0.00	0.54	-38.7 ± 12.5	-52.31
Null (intercept)	3	122.45	1.83	0.22	117.8 ± 22.3	-56.22
SVL_{mean}	4	123.98	3.36	0.10	-14.9 ± 7.0	-53.99
T_{max}	4	124.55	3.93	0.08	11.2 ± 5.7	s-54.27
Altitude	4	125.50	4.88	0.05	0.22 ± 013	-54.75
T_{min}<br>+<br>log(T_{min})	5	128.15	7.53	0.01	204.1 ± 230.9<br>-2515.7 ± 2389.6	-51.57
SVL_{mean}<br>+<br>log(SVL_{mean})	5	132.58	11.97	0.00	-162.0 ± 276.4<br>6996.4 ± 13143.4	-53.79
Altitude<br>+<br>log(altitude)	5	133.40	12.78	0.00	-2.5 ± 3.0<br>3385.8 ± 3754.1	-54.20
T_{max}<br>+<br>log(T_{max})	5	133.43	12.81	0.00	-18.2 ± 100.2<br>974.4 ± 3312.2	-54.21

k : number of parameters, ΔAICc: difference with AICc of the best model, w_i: model likelihood.

Supplementary table S4. Principal component analysis (PCA) including the main determinants of population collapse. Environmental variables included in the PCA were previously selected based on their relationships (see Table S1), including the relative change in abundance (Δabundance**)**, the minimal temperature (T_{min}), and the altitude. Table shows the eigenvalue of the first 3 axes (PC_1 to PC_3), percentage of variance explained, and the inertia of each variables on the first two axes.

		PCA axes		
		PC_1	PC_2	PC_3
Eigenvalue		2.08	0.67	0.24
Variance		69.5	22.5	8.1
Inertia	Δabundance	4029	374	-
	T_{min}	3540	2455	-
	Altitude	2431	7171	-

Supplementary Figure S1. Annotated TRF gel to measure telomeric DNA length in the Common lizard. Smears were obtained with a pulse-field gel electrophoresis on a 0.8% agarose gel. Lanes 1, 16 and 29 contain size marker and lane 30 was used for blank. Note that in this gel, intra-gel coefficient of variation was calculated by repeating 3 samples in lanes 2 to 4 and in lanes 26 to 28 (also measured in the 4 other gels to assess the inter-gel coefficient of variation).

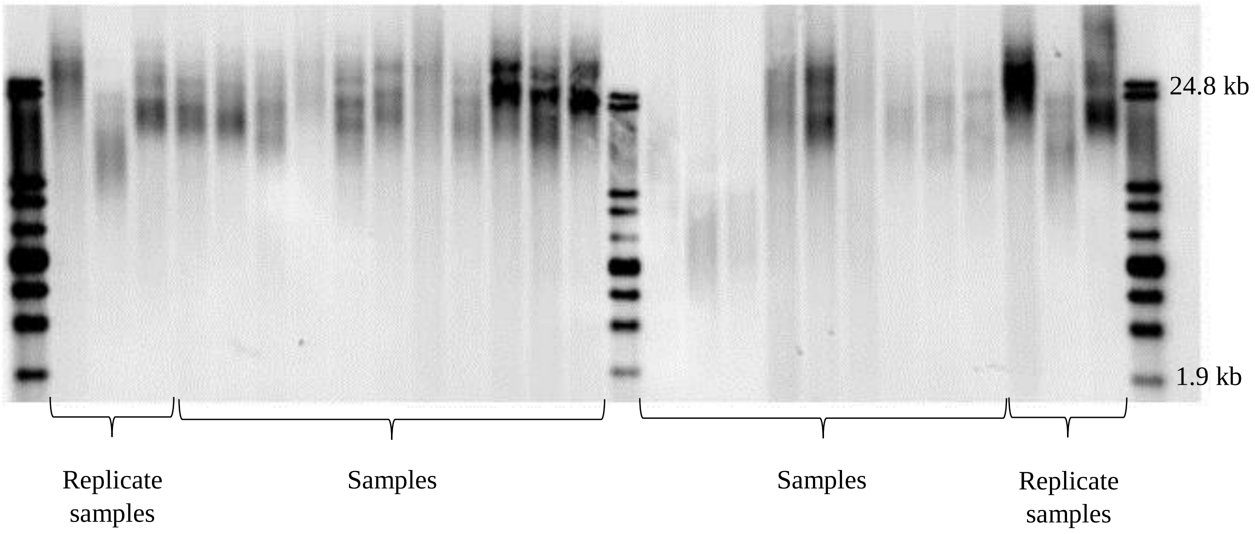

Supplement: Supplementary file 1 — Supplementary information [file 41598_2017_17323_MOESM1_ESM.pdf]
